# “It’s a big, round circle of knowledge to me. I don’t know how else to explain it”: strengthening cultural identity through Indigenous placekeeping and food sovereignty practices in Kingston, Ontario

**DOI:** 10.1177/11771801251342662

**Published:** 2025-06-08

**Authors:** Kaitlyn Patterson, Sherri Dutton, Autumn Watson, Jessica Pace, Penni-Dawn Kernot, Constance Carriere-Prill, Amanda Wilson, Jennifer Kehoe, Sheldon Traviss, Imaan Bayoumi, Eva Purkey, Colleen Davison

**Affiliations:** 1 Department of Public Health Sciences, Queen’s University, Canada; 2 Dalla Lana School of Public Health, University of Toronto, Canada; 3 Indigenous Diabetes Health Circle, Canada; 4 Oversight Committee, Canada; 5 Kingston Native Centre and Language Nest, Canada; 6 Grassroots Indigenous Youth Initiatives, Canada; 7 Department of Family Medicine, Queen’s University, Canada

**Keywords:** cultural identity, Indigenous food sovereignty, Indigenous health, Indigenous placekeeping

## Abstract

Indigenous identity is supported by connections to land, community, and culture and is a key aspect of health. Cultural identity is threatened by settler colonialism, and urban Indigenous Peoples experience unique challenges including the myth that Indigeneity is incompatible with urban areas, even though most Indigenous Peoples in Canada live in cities, and many express their identities through urban Indigenous food sovereignty (IFS) practices. Kingston Native Centre and Language Nest, Indigenous Diabetes Health Circle, and Queen’s University researchers created IFS programming in Kingston, Ontario, Canada, to support wholistic health. Impacts on Indigenous participants’ cultural identities were explored through one-on-one interviews (*n* = 11), one programme participant sharing circle (*n* = 8), and one programme facilitator sharing circle (*n* = 12). Ribbon skirt analysis with storytelling methodology produced storied results. Findings demonstrate how participants express their cultural identities within processes conceptualized as Indigenous placekeeping through IFS practices that benefit wellbeing.

## Introduction

For many Indigenous Peoples, a clear sense of place-based cultural identity is critical to health and wellbeing (Barker et al., 2017; Lin et al., 2023; Monchalin et al., 2020). Connections to place are made by long-standing relationships or kinships, transmitted knowledge and stories, and everyday practices in relation to land, water, animals, plants, and spirit; connection to place frames identity and illustrates belonging (Daigle, 2024). However, Indigenous places and lifeways that shape cultural identity have been impacted by settler colonialism in Canada including by imposed residential schools, reserves and settlements, resource extraction, and identity legislation (Big-Canoe & Richmond, 2014; Nightingale & Richmond, 2022a, 2022b; Richmond & Ross, 2009). Indigenous Peoples living in urban areas face unique barriers to their cultural identities such as the pervasive myth that Indigeneity is incompatible with cities (Dorries, 2023; K. Wilson & Peters, 2005). This belief stems from government policies that attempt to confine Indigenous culture to isolated spaces such as reserves and stereotypes that tie Indigeneity solely to *nature* or relegate it to pre-contact (Gilio-Whitaker, 2019; Lawrence, 2004; Palmater, 2011). Indigenous Peoples who move to cities are thought to have abandoned their communities, cultures, and identities to join mainstream society (Congress of Aboriginal Peoples, 2019). They are positioned as *out-of-place* or *less authentic* which serves to erase Indigenous identities, histories, knowledges, and self-determination within urban spaces (Coulthard, 2014; Nursey-Bray et al., 2022).

Yet, contrary to colonial narratives, the majority of Indigenous Peoples across Canada live in urban centres (Statistics Canada, 2016). Resulting from colonialism, many Indigenous Peoples have relocated from their ancestral territories and formed new connections within urban places (Cidro et al., 2015). Because of this movement, and because all cities exist on Indigenous lands, urban environments are interwoven with Indigenous lifeways; they coexist alongside, against, and within Indigenous relational networks (Dorries et al., 2019). Urban Indigenous communities are held as, and (re)made into, significant places of cultural identity where people connect with land, community, and culture in support of health and wellbeing.

One way urban Indigenous cultural identities have been expressed is through the growing Indigenous food sovereignty (IFS) movement (Cidro et al., 2015; Mundel & Chapman, 2010; Neufeld et al., 2020; Peach et al., 2020). IFS involves Indigenous Peoples maintaining and strengthening their relationships with land and waters through traditional, localized, and modern practices that provide healthy, culturally meaningful, and lasting food sources (Alabi & Robin, 2022; Miltenburg et al., 2022; Morrison, 2011). IFS recognizes food as sacred and affirms Indigenous Peoples’ long-standing responsibilities to care for the land as members of Creation. IFS requires active participation in food systems, which often demands transformation of colonial laws and policies largely opposed to Indigenous lifeways (Morrison, 2011). IFS is a conceptual framework supportive of Indigenous health, encompassing culture, language, self-determination, and identity (Coté, 2016; Ray et al., 2019).

Within Katarokwi—greater Kingston, Ontario, Canada—members of the urban Indigenous community recognize the impact of IFS on cultural identity and health. Katarokwi is on the traditional territories of three large Indigenous cultural groups: the Anishinaabeg (an Indigenous People, Great Lakes Region, North America), Haudenosaunee (an Indigenous People, Ontario and Quebec, Canada, and New York, USA), and Huron-Wendat (an Indigenous People, St. Lawrence Valley up to Great Lakes Region, North America). It is home for approximately 7,000 Indigenous Peoples from three groups recognized in the Canadian *Constitution*: First Nations, Métis (one of three groups of Indigenous Peoples, recognized in the Canadian Constitution, Canada), and Inuit (an Indigenous people of Arctic Canada), (Statistics Canada, 2022). The Anishinaabeg, Haudenosaunee, and Huron-Wendat encompass many First Nations communities and peoples over wide geographies in North America. Previous community-based participatory research led by the Indigenous Diabetes Health Circle (IDHC) and Queen’s University researchers identified local IFS initiatives as having a positive impact on the overall health and wellbeing of the Katarokwi urban Indigenous community (Watson et al., 2022). With these results, IDHC and Queen’s University researchers partnered with Kingston Indigenous Languages Nest (KILN)—which is now the Kingston Native Centre and Language Nest (KNCLN)—to create the Aki Gimiinigonaa Mshkooziiwin (The Land Gives Us Strength) Project (Aki Project). In July 2023, KILN was ratified as a member of the Ontario Federation of Indigenous Friendship Centres and changed its name to KNCLN. The Aki Project provided funding for IFS programming facilitated by KNCLN from June 2022 to June 2023. IFS programming harnessed community strengths to support cultural identity, community and land-based connections, and mental, emotional, physical, and spiritual wellbeing. Programming involved three streams: foraging (33 events), gardening (28 events), and building personal bundles through cultural activities (52 events) including fire keeping, language learning, smudging, ribbon skirt and ribbon shirt making, rattle making, animal harvesting, and hide workshops. While impacts of Aki Project programming are manifold, this article focuses on how Indigenous participants conceptualized their cultural identities alongside influences of land, community, and culture during the Aki Project.

## Methods

This community-based participatory research project was developed from long-standing relationships and is guided by principles of ownership, control, access, and possession (First Nations Information Governance Centre, 2014). The project was created by KNCLN, IDHC, and Queen’s University researchers. KP and SD are Katarokwi Indigenous community members and joined the project to support data analysis and write-up. The Oversight Committee (OC) consists of additional Katarokwi Indigenous community members ST, JK, AW, and PDK who provided feedback on analyses and dissemination.

Data collection was led by team member AW and involved 15 semi-structured one-on-one interviews with Aki Project programme participants, as well as one Indigenous Community Circle (ICC) of programme participants (*n* = 15) and one Front Line Worker Circle (FLWC) of programme facilitators (*n* = 15). No demographic data were collected, although 31 participants self-identified as Indigenous (11 interviewees, eight ICCs, 12 FLWCs).

**Figure 1.**
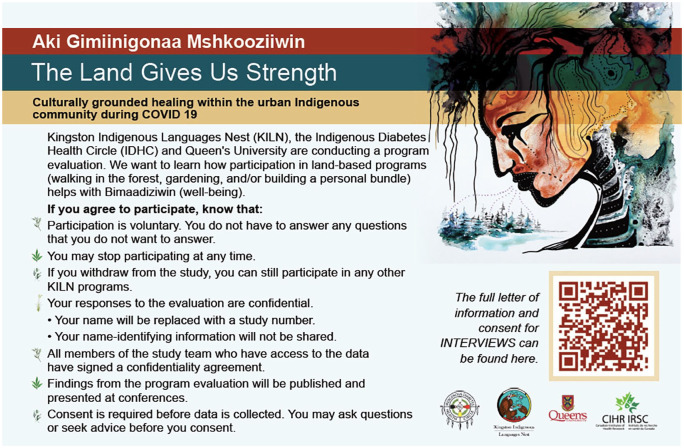
Consent postcard.

Programme participants were invited to join an interview or circle in-person during programming and by social media posters. Programme facilitators were invited by e-mail. Data collection occurred Fall 2022 through Winter 2023 alongside programming. Interviews ranged from 16 to 60 min in length, while the ICC was 110 min and the FLWC was 137 min. All participants provided verbal consent facilitated by postcard (Figure 1) and were offered a smudge—a ceremony involving the washing of the body with the smoke of various burned medicines including sage, tobacco, cedar, and sweetgrass—and given semaa (tobacco) and a CAN $25 gift card. Interviews and circles incorporated local Indigenous protocols and a Knowledge Holder was available for debriefing with participants.

Interview and ICC questions explored participants’ experiences with land and community connections, cultural identity, and wellbeing in the context of Aki programming. The FLWC involved these same elements, along with facilitators, barriers, and sustainability of IFS programming in Katarokwi. All interviews and circles were conducted in English. They were audio recorded and transcribed verbatim prior to analysis. This project received ethical clearance from the Queen’s University Health Sciences and Affiliated Teaching Hospitals Research Ethics Board.

## Data analysis

Team members KP, SD, AW, and JP reviewed all transcripts and completed high-level coding with NVivo 14 (Clarke & Braun, 2017; QSR International, 2023). Preliminary results were presented to an OC consisting of Katarokwi Indigenous community members JK, ST, AW, and PDK, and this article’s topic was supported.

Using a Ribbon Skirt framework of data analysis, compiled team coding and combined reflexive thematic and storytelling analysis to create storied results (Patterson et al., 2023). With ribbon skirt analysis, storytelling is a lens through which to see the world; stories carry histories and cultures that shape past, present, and future (Loppie, 2007; Thomas, 2015). Stories are contextual, subjective, and often collaborative processes of creating knowledge or revealing intricate, multiple realities by examining experience in relation, wherein all interest holders are involved in meaning-making (Archibald, 2008). Storytelling invites reflexivity, whereby authors and readers examine their intentions and positionality as they co-create meaning (McGuire-Adams, 2023). Within research, stories are meaningful when they reflect the experiences and perspectives of participants, as opposed to other approaches that seek to identify a universal truth (S. Wilson, 2008).

Within Anishinaabe (an Indigenous person from the Great Lakes Region, North America) culture, ribbon skirts are beautiful works of creativity, connection, and strength. Ribbons typically encircle the skirt, representing the cyclical nature of stories and Mother Earth. Stitches that attach ribbons to the skirt represent interpretive storytelling and demonstrate the connections of researchers to the research. The Ribbon Skirt framework resembles other methodologies developed by Indigenous researchers including the Herringbone Stitch Model (Andrews, 2020), the Story Rug framework (Tachine, 2015), and a Butterfly framework for coding (Chiblow, 2021). Utilizing Indigenous methodologies contributes to decolonizing research processes and supports Indigenous self-determination (Smith, 1999).

Ribbon skirt analysis was used in this project because it stitches varied knowledges and experiences together to produce genuine stories when researchers have strong connections with participants and their community(ies) (Patterson et al., 2023). Given their pre-existing connections with the Katarokwi Indigenous community, KP and SD led analysis and iteratively wove together data sources into a cohesive story that centred on participants’ transcripts. Although all interview and circle data was reviewed, only data from self-identified Indigenous participants were used within this article. Meaning-making was informed by relevant literatures and researchers’ positionality, including KP’s and SD’s personal, experiential, spiritual, and cultural influences as Anishinaabe women. KP and SD engaged in critical reflection throughout the storytelling process and incorporated feedback from the OC and research team. Similar to ribbon skirts, the story is meant to demonstrate community strength for community benefit (Patterson et al., 2023).

In the storied results, all dialogue consists of participant quotes except for bracketed text which was added to improve flow while remaining aligned with participants’ contributions. Participants are represented by pseudonyms in the form of seven fictional characters: Amik, Makwa, Waawaashkeshi, Binesi, Gaagaagi, Miskwaadesi, and Giigoonh. Dialogue from characters often includes quotes from multiple participants; participant interview quotes are differentiated alpha-numerically at the end of each participant quote, for example, as P37, while participant contributions from sharing circles are differentiated by respective FLWC and ICC acronyms. A single character quote in the story sometimes includes quotes from multiple participants; differentiations are likewise made alpha-numerically at the end of each participant quote. While KP and SD offer key findings and analyses, many aspects of the story are open to interpretation as per storytelling methodology (Kovach, 2009).

## Storied results

I feel the heat from the July sun beat down on my head and shoulders. It is another blistering day in Katarokwi and I’m making my way down Montreal Street towards KNCLN. Lately, the Native Centre has been running more land-based programmes around town. There have been a few get-togethers every month this summer and I’ve been to almost all of them. Today, a group is meeting before we head to the IFS garden on Highway 15 for a few hours.

Reaching the Native Centre, I take a left and plod up the grass, heading for the pastel yellow door that marks the entrance of the limestone building. I knock three times and wait to be welcomed in. As I do, I hear others coming up behind me.

Amik, Makwa, and Waawaashkeshi walk over from up the street, and Binesi and Gaagaagi hop out of their car and head over from the parking lot. They all have their gardening clothes on—worn t-shirts, shorts, and mud-caked runners.

“[Boozhoo (Hello), Miskwaadesi!]” Amik waives at me.

“[Aaniin (Hello)]”, says Makwa, heading toward me. She gives me a nod before plopping down on the grass.

Waawaashkeshi takes a seat next to Makwa. “[I’m looking forward to getting out there today]”, Waawaashkeshi announces. “I usually go out just to clear my head and stuff like that—to see what I can find out there… But there’s not very much to do in the city. I come from … northern Ontario”. (P34)

“[Where are you from?]” I ask. I don’t know Waawaashkeshi very well. I met him on the last garden day, but we didn’t get a chance to talk.

“I don’t really know”, Waawaashkeshi says. “I just know my dad was full blooded. And it’s kind of hard to tell what you are when you don’t really know yourself”. (P34)

“[You’re not alone in that uncertainty]”, Amik says. “Connecting to my Native side—I haven’t had a lot of that connection. It was always kind of like a secret, almost, in our family. It wasn’t spoken about. I was lost and really didn’t understand who I was and felt like a piece of me was missing (P36). And now that I’ve been able to understand and get those teachings, I don’t have a piece of me missing anymore”. (P37)

“Now”, Amik continues, “I want to learn [the culture] so that I can pass it on to my nieces and nephews . . . I want to be able to pass it on to them, so that they know where they came from and our connection to the land (P36). Teaching the little ones about our medicines at a young age—and our language (ICC). It’s a good thing because it helps kids learn. It helps them become strong inside (ICC). It lets them know that they have the right to feel their sense of belonging—of who they are and where they’re from and what they can do”. (ICC)

“That’s what this place is about”, (P46) says Makwa. “[It’s] where our young ones, like my granddaughter, can learn the whole spectrum of who they are within their culture (ICC). This community seems to create family. And that helps the generations that come behind that individual”. (ICC)

It has been a while, so I knock again on the door. Moments later, the door swings open and Giigoonh steps out, excited and smiling.

“[Boozhoo]!” Giigoonh beams, ushering all of us inside.

I smile and we follow her in where the air conditioning offers a break from the city heat.

The six of us wait inside the main area, while Giigoonh runs around upstairs, gathering a few things before we all pile into two cars to go to the garden. The garden is out on the east end of Kingston, about a 10-min drive from the Native Centre, and a good walk from the closest bus stop. I don’t have a car, so it’s been nice getting a ride from Giigoonh all summer.

The Native Centre’s gardens at City Park and Tipi Moza are way closer to my place downtown. I remember when they started the City Park garden. The first year there were four planters—each a colour of the Medicine Wheel, holding sacred medicines and offering a gathering place. It’s hard to believe that a statue of Canada’s first prime minister once stood where our garden now sits. It’s been powerful to see the community reclaim that space—our site of reclamation in the heart of the city.

As we wait for Giigoonh, I wander over to the Centre’s lobby to check out the food sharing shelf where baskets of food wait for pick up. Next to the shelf is a cabinet full of medicines for community members: semaa, giizhik (cedar), maskodewashk (sage), and wiingashk (sweet grass)—all ready for ceremony. Decorating the cabinet are community event flyers: drumming, full moon ceremony, and rattle workshops. Under the front window is another sharing station stocked with tampons, diapers, and baby wipes.

Suddenly, I hear Giigoonh on the stairs, coming down to join us with four, full tote bags hung on her arms, stuffed with row covering, gloves, and seeds for the garden. I grab a few bags from her, and we all follow Giigoonh out to the parking lot, splitting ourselves into two cars before heading to the garden.

Out on the land, before we start our work, we gather in circle and share a smudge—a ceremony where we burn medicines like maskodewashk to produce smoke that we use to wash our bodies of negativity. Then Giigoonh goes around and hands out semaa.

“When I’m going to harvest”, shares Giigoonh, “The most important thing to do is to always give thanks. And you want to put your semaa down to be able to . . . you’re giving thanks. You’re connecting. You’re thanking the plant for its life and for giving you life (P36). [And when we smudge], we’re cleansing ourselves, purifying ourselves”. (P53)

With semaa in our palms, we each search for a spot on the land to lay down our medicine. Surveying the space and considering my options, my eyes drift across the field to where we planted mitigoog (trees) last fall. Before then, the field was mostly hay. As I reminisce, Amik comes up beside me to lay their semaa too.

“[Amik, I remember when] I could just hear the cars going by”, I say. “And the wind was so loud we couldn’t hear each other very well. And it didn’t have the kind of warmth—and now, the trees, I’ve seen them grow. And I can feel it’s becoming more and more welcoming”. (P62)

Amik smiles and we put our semaa down.

Standing back up, I glance around to see what others are up to. Makwa and Giigoonh are searching for ripe odatagaagominag (blackberries), while Waawaashkeshi, Binesi, and Gaagaagi savour a few more sips of coffee.

Amik jumps to work and asks me, “[Will you help me with the tomatoes—er, the gichi-oginiig (tomatoes)?]” They glance over at the Anishinaabemowin (Anishinaabe language) label at the front of the bed to make sure they’ve got it right.

I call out to the others for help—with two, long gichi-ogin (tomato) beds, there’s lots of work to do. A few minutes later, the others join us, and we begin our work staking, suckering, and thinning the gichi-oginiig.

“[Hey, anyone make it out to the foraging and rabbit harvesting workshops last week? How’d they go]?” I ask everyone while I trim some undergrowth.

“[Yeah, I went]”, answers Binesi. “[They were great]. Like, we’re doing the rabbit snaring, okay? That teaches the women, and the children, and the men—look, you can [make] your own food. You can gather your own food”. (P53)

“To me, it’s a better way of living all around”, Amik adds. “Because we’re not just working on the mental aspect, or the physical—we’re bringing it all up at the same time . . . Because if you bring up the person as a whole, they become a better person . . . And it helps, because we get to find out who we are, where we come from”. (P46)

“[I think having good teachers is a part of that]”, Gaagaagi offers. “You know, [Nookomis Nigig] can teach me the language for a plant, but I wouldn’t necessarily know what to do with it . . . But she gets her hands into the work, and she shows people how to work with our plants. She shows people how to integrate the language. And I think it’s that step-by-step modeling of the teachings that she’s learned . . . That is something that people can then grab hold of and walk with . . . Something in action can translate to further action”. (FLWC)

“I hope that Grandmothers will continue to guide and support us because we’ll be Grandmothers someday as well”, says Giigoonh. “And then we’ll do the same thing for the next ones coming up—the young ones, the little ones that aren’t even here yet. I’m just real grateful for Grandmothers in my life, and Aunties in my life”. (FLWC)

“[We create] a sense of community”, Binessi agrees. “Sharing food, sharing knowledge, sharing help . . . because that’s the way community works and that’s the way, I find, the culture works. You’re learning the way food connects you. You’re learning . . . how you can look at everything. It’s a big round circle of knowledge to me. I don’t know how else to explain it (P36). We get to find out who we are”. (P46)

“[When I think about who I am]”, I reflect, “[It’s me] being true to my roots—my Ancestors—while also sharing with others who are a different culture. And learning their culture at the same time. Because then I become a more well-rounded person . . . And it’s the Medicine Wheel that teaches us we are all here and we all need to bring something to the table”. (P37)

“[But I also think] what are the Western, very catholic-based things that have gotten mixed in with our teachings?” Gaagaagi asks. “Like the ribbon skirt one—[being told to wear a ribbon skirt at certain ceremonies]. It’s hard to move in a skirt sometimes. It’s not going to be practical all the time”. (FLWC)

Reflecting on Gaagaagi’s words, I consider my own background and all the influences in my life that shape who I am. As I ponder, I pop another juicy gichi-ogin from the vine and set it beside me.

“[Doing what we do in community] can sometimes be really difficult”, Giigoonh says. “But we just have to keep moving, keep pushing forward and keep doing what we do. And everything we do, we do it for the people . . . That helps keep us in check, and keeps moving things forward. So we can always bring . . . good things to the community that desperately needs it. Not because of the community itself, but because of everything that has happened over the past 500 years that has tried to erase us, and disappear us, and silence us, and break us apart amongst each other . . . That is what the community is feeling and this is not new to community. It’s a lot of urban spaces that feel that”. (FLWC)

Binesi nods at Amik and says, “I feel like the kids—they’re craving . . . they thrive for community connection . . . And I think the way to do that is to [have] more cultural ceremonies and build it off of the way things used to be. Going fishing when it’s time to go fishing. Going hunting when it’s time to go hunting” (FLWC).

“For a person to have a connection with the land . . . it’s one of the most important things”, (FLWC) Gaagaagi affirms. “I feel connected to the land where I grew up . . . We have a camp and I go for a couple of weeks at least every year. And that land and water I feel so connected to because for generations, and generations, and generations that’s where my family is from . . . And here in Kingston—it’s interesting being an urban community, right? To develop another strong connection to the land . . . I’ve only been here a few years, so I’m still developing that. But this community, I think the idea of building that space, like Highway 15, and having community come—I’ve planted some trees with my grandson and I know there’s medicines there. I think that’s a great thing for an urban community to have: land—physical land—and blue skies, and birds, and plants, and soil, and all of that to connect us to where we meet and do things together. I think that’s what a lot of us who aren’t from here need (P62). We need to keep our land so that we can have a good life”. (ICC)

“My connection with the land is . . . the land is everywhere”, Makwa says. “I’m comfortable doing what I need to do on the land, going down in the little green space next to the industrial park . . . Most of my [ceremonial] fire keeping gathering happens within Kingston city limits . . . So, I never felt like I had a loss of connection with the land because it’s always around me”. (P60)

“[For sure]”, Giigoonh agrees. “I get really excited when I go foraging [in town]. I’m always like, ‘Hey, friend of mine, do you want to eat this random dish I’ve made?’ (FLWC) [But] living in an urban Indigenous setting, we don’t have access to a lot of land—accessible land”. (FLWC)

“But [KILN–now the Native Centre] is starting to create that space for people”, (FLWC) Makwa offers.

“[And cool events]”, adds Waawaashkeshi. “One of the activities I did was to learn how to make a sacred fire. I got to light a fire with flint. I was quite proud of that . . . And when it lights, and the fire comes up—that’s just amazing. It lifts my spirit completely”. (P36)

Buzz buzz buzz. I hear a vibration coming from Giigoonh’s pocket. Giigoonh reaches down, grabs her phone from her pocket, and checks her messages.

“[It’s Nookomis Nigig . . . She wants us to meet her on Belle Island across the bridge for some buckhorn removal. Wanna go?]” Giigoonh reports.

The gichi-oginiig are looking better, so we all agree to go. We put our tools back in the shed, toss the pruned greenery in the compost, place our harvested gichi-oginiig in buckets, and pile back into our cars.

In the backseat, I think about our time today on the land. What does this place do for community? How have we impacted this space? What have the gichi-ogiinig taught me? What will I learn from Nookomis Nigig and the buckthorn on Belle Island? Or from the maskodewashk and wiingashk at City Park and Tipi Moza? I let out a big sigh and rest my head against the car window. On the other side of the glass, I watch the green and grey of the city rush by as we head west towards the island. As our responsibilities move from one space in the city to another, we follow, bringing community with us and greeting it when we arrive—a diverse, wholistic, relational entity in constant motion.

## Limitations

Interpretation was limited from a lack of demographic data. Participants who voluntarily self-identified as Indigenous were included in analysis, which may have resulted in missed contributions. Sharing circle participants were not assigned unique IDs during transcription, which impacted participant quote reporting. However, given that data collection and analysis were led by several team members with community connections—including KP, SD, JP, and AW—it was possible to differentiate participants. Data collection ended Winter 2023 and, therefore, missed more recent Aki Project activities including KNCLN garden expansions. Finally, Anishinaabemowin is included in the story given KP’s and SD’s Anishinaabe identities, and ideally more languages would have been incorporated to better represent Katarokwi Indigenous community.

## Discussion

Participants described the strengths of their cultural identities, while also highlighting challenges they face in an urban setting. Notably, in many instances, participants described experiences of environmental dispossession—separations from ancestral lands and communities as a result of colonialism (Nightingale & Richmond, 2022). In this context, several participants acknowledged one major challenge to their Indigeneity being limited access to land and safe gathering spaces in town. This hindered their ability to practice their cultures by growing food and medicines, language learning with community, or participating in land-based ceremonies. Overcoming this barrier, some participants are able to return to ancestral territories or connect with land in small areas throughout the city, for example, in backyards and parks. Several participants also found the Native Centre and Aki Project helpful in facilitating connections to land and community.

Some participants also cited a lack of cultural and traditional ecological knowledge as a barrier to strengthening cultural identity. In response, many participants emphasized the value of receiving teachings from Knowledge Holders and Grandmothers within and outside the Aki Project. The importance of intergenerational knowledge transfer for individual and community wellbeing is widely recognized (McGuire-Adams, 2023). Indigenous women, and Grandmothers in particular, are acknowledged for strengthening their communities and championing the IFS movement on Turtle Island—another name for North America. Indigenous women are woven throughout the fabric of community and are recognized for their leadership and knowledge that foster community resilience (S. Maracle et al., 2020; Neufeld et al., 2020; Waaskone Giizhigook Ray, 2023).

Some participants also acknowledged harmful patriarchal and heteronormative ideologies that prescribe gender identities for community members. Participants resist these influences by discussing and expressing their identities in authentic, practical ways. Similarly, across Turtle Island, Indigenous Peoples have called out racism, misogyny, homophobia, and transphobia that inflict harm on, in particular, Indigenous girls, women, and Two-Spirit and gender-diverse people (National Inquiry into Missing and Murdered Indigenous Women and Girls, 2019). These influences have been challenged across decades of activism, while Indigenous Peoples have asserted their ancestral lifeways and *come in* to their authentic identities in support of their wellbeing (Simpson, 2011; A. Wilson, 2018).

Despite challenges, participants described the strengths and expressions of their Indigeneity. When describing their cultural identities, many participants discussed their everyday activities: the cultural practices they engage in routinely, with whom they practice, and where they go at which times of the year or life cycle. Participants also described the teachings they receive from Ancestors, family, and mentors that guide their actions, and how they pass these teachings on to younger generations. Through the Aki Project, participants engaged in foraging, gardening, and building personal bundles. Many participants also connected with community cultural practices that ran parallel to the Aki Project including language groups and ceremonies. Their cultural practices were influenced by kinships and community ties, along with their relationships with ancestral homelands and Katarokwi. The ways in which participants discussed their identities demonstrates Indigeneity as a process—ongoing actions in connection with others in particular places that contribute to who they are as Indigenous Peoples.

The idea of identity as a process is affirmed by Indigenous scholars including Lyons (2010), who describes Indigenous identity as a social process, or a communal construction of meaning, that is historically, politically, and materially influenced. As Lyons (2010) states, “Indian identity is something people do, not what they are” (p. 40). Likewise, G. Maracle (2021) notes, “Our identities are active processes that are affirmed, supported and enhanced through communities and relations”, and these processes are constantly renewed (p. 18). Relatedly, Holm et al. (2003) theorize identity through the concept of *peoplehood*, framing it as a dynamic, interacting balance of sacred history, ceremonial cycles, language, and ancestral homelands. Weaving through this framework is the importance of maintaining respectful relationships with all living beings as a core component of an Indigenous cultural ideal (Alfred & Corntassel, 2005).

Indigenous Peoples’ histories, ceremonies, languages, and lands have been threatened by colonialism, but communities actively strengthen and remember their identities through “daily acts of renewal” through practices such as storytelling, cultivating food, or language learning, and these everyday actions are the “foundations of resurgence” (Corntassel, 2012, p. 89). As Simpson (2017) describes, “How we live, how we organize, how we engage in the world—the process—not only frames the outcome, it is the transformation” (p. 19). Guiding Indigenous resurgence that strengthens identity are the place-based practices and knowledges that teach people how to interact with one another and the land in respectful, reciprocal ways that uphold good relationships (Coulthard, 2014).

Within Indigenous health research, and in urban spaces in particular, cultural identity restoration is being increasingly explored through the concept of Indigenous placekeeping, or place-making, which describes Indigenous Peoples engaging in complex and reciprocal relationships with lands, waters, and all living beings within a space they are directly embedded and responsible to. Indigenous placekeeping implicates Indigenous Peoples as both determining actors and dependent beings that are formed by their surroundings as they contribute to and transform urban ecologies (Hatala et al., 2019; Latulippe et al., 2023; Miltenburg et al., 2023).

Indigenous placekeeping insists that land is sacred and exists everywhere, thereby challenging the idea that *nature* is only *out there*, beyond city limits (Engle et al., 2022; Gilio-Whitaker, 2019; K. Wilson & Peters, 2005). Similarly, Indigenous relationships with land are everywhere, extending across imposed colonial demarcations including national borders or city limits, and often occupying multiple positions such as reserve lands and urban places (Dorries, 2023; Nursey-Bray et al., 2022). Through a lens of Indigenous placekeeping, urban Indigenous Peoples’ cultural identities are strengthened by their connections to multiple places, inclusive of their ancestral lands and the cities they call home (Daigle, 2024).

Within the Aki Project, participants articulated their identities as being lived out through everyday activities, or resurgent processes, which can be understood as Indigenous placekeeping through a pathway of IFS initiatives in Katarokwi. As they lived out and strengthened their cultural identities through wholistic engagement—in other words, through mental, emotional, spiritual, physical, social, geographical connections, as many participants described, they actively contributed to their health and wellbeing. By engaging in IFS practices within the Aki Project, their cultural identities were shaped in a circular process of placekeeping, open for continual renewal.

## Conclusion

Many Indigenous Peoples experience cultural identity uncertainty when their connections to land, community, and culture are disrupted by colonialism. While community strengths and strategies take many forms, Indigenous community-based programming offers one way to support cultural identity. Within and alongside the project, participants lived out their Indigeneity in relational and place-based ways. Conceptualized as Indigenous placekeeping through IFS activities, many participants strengthened their cultural identities through ongoing acts of resurgence in cyclical processes that benefitted wholistic health and wellbeing.
